# MicroRNA-181 Regulates CARM1 and Histone Aginine Methylation to Promote Differentiation of Human Embryonic Stem Cells

**DOI:** 10.1371/journal.pone.0053146

**Published:** 2013-01-03

**Authors:** Zhenyu Xu, Junfeng Jiang, Chen Xu, Yue Wang, Lei Sun, Xiaocan Guo, Houqi Liu

**Affiliations:** Research Center of Developmental Biology, Second Military Medical University, Shanghai, China; Wellcome Trust Centre for Stem Cell Research, United Kingdom

## Abstract

As a novel epigenetic mechanism, histone H3 methylation at R17 and R26, which is mainly catalyzed by coactivator-associated protein arginine methyltransferase 1 (CARM1), has been reported to modulate the transcription of key pluripotency factors and to regulate pluripotency in mouse embryos and mouse embryonic stem cells (mESCs) in previous studies. However, the role of CARM1 in human embryonic stem cells (hESCs) and the regulatory mechanism that controls *CARM1* expression during ESCs differentiation are presently unknown. Here, we demonstrate that CARM1 plays an active role in the resistance to differentiation in hESCs by regulating pluripotency genes in response to BMP4. In a functional screen, we identified the miR-181 family as a regulator of *CARM1* that is induced during ESC differentiation and show that endogenous miR-181c represses the expression of *CARM1*. Depletion of CARM1 or enforced expression of miR-181c inhibits the expression of pluripotency genes and induces differentiation independent of BMP4, whereas overexpression of *CARM1* or miR-181c inhibitor elevates Nanog and impedes differentiation. Furthermore, expression of *CARM1* rescue constructs inhibits the effect of miR-181c overexpression in promoting differentiation. Taken together, our findings demonstrate the importance of a miR-181c-CARM1 pathway in regulating the differentiation of hESCs.

## Introduction

Human embryonic stem cells (hESCs) are valuable resources for clinical therapies as well as biological and pharmacological research due to their pluripotency and unlimited self-renewal ability [1]. The maintenance of ES cell self-renewal is controlled by a network of transcription factors, including Oct4, Sox2 and Nanog [2], [3], [4]. Any perturbation of these factors collapses the self-renewal circuitry and triggers specific or mixed lineage differentiation [5]. The regulatory mechanism of these “core” pluripotency factors is therefore critical in the study of hESCs [1].

Recently, the role of chromatin structure and epigenetic modifications in controlling gene expression during ES cell self-renewal and differentiation has been under intense investigation [6], [7], [8], [9]. Changes in chromatin structure are mediated through chemical modification of histones (e.g. acetylation, methylation, demethylation, and ubiquitination), DNA methylation and the action of DNA-binding proteins and chromatin-remodeling enzyme complexes [6], [9], [10], [11], [12]. However, while histone lysine methylation and the machinery involved in this process have been extensively investigated in the maintenance of human ES cell self-renewal, differentiation and somatic cell reprogramming [10], there exists little complementary information regarding the role of histone arginine methylations in differentiation and lineage determination. Recent studies indicate that histone H3 methylation at R17 and R26, catalyzed by coactivator-associated protein arginine methyltransferase 1 (CARM1, also known as PRMT4) modulates the pluripotency in mouse embryos and mouse embryonic stem cells (mESCs) [13], [14]. This raises the question of whether CARM1 and histone arginine methylation may similarly modulate the pluripotency of hESCs.

The regulation of *CARM1* expression in hESCs, especially the regulation on the epigenetic level, also remains undefined. MicroRNAs (miRNAs), which are evolutionarily conserved noncoding RNAs with a length of 18–24 nucleotides, have been shown to play critical roles in the regulation of gene expression and multiple cellular processes [15], [16], [17]. Through base pairing with mRNAs at partially or fully complementary sites, miRNAs induce mRNA cleavage or translational repression [18]. The cell type-specific expression signature of miRNAs in ESCs has been used successfully to distinguish ESCs from differentiated cell types [19], [20], [21], [22], [23]. More than 100 miRNAs are differentially expressed in hESCs and the differentiated embryoid bodies (EBs) [24]. However, whether *CARM1* expression and histone arginine methylation are regulated by miRNAs is currently unknown. The characterization of *CARM1*-targeting miRNAs and their underlying molecular mechanisms is of great importance to understand the regulatory mechanism of histone arginine methylation during ESC self-renewal and differentiation.

Herein, we demonstrate that CARM1 plays an active role in resisting the differentiation of hESCs. After knockdown of *CARM1*, the expression of pluripotency genes decreased and hESCs entered differentiation programs. By contrast, overexpression of *CARM1* increased the Nanog expression level as well as the resistance of hESCs to differentiation cues. We also found that *CARM1* was post-transcriptionally regulated and was directly targeted by miR-181, which represses the 3′ untranslated region (3′UTR) of *CARM1* in hESCs. Overexpression of miR-181c promoted the differentiation of hESCs, whereas overexpression of miR-181c inhibitor impeded differentiation. Furthermore, CARM1 partly rescued the effects of miR-181c expression by elevating the transcript levels of *Nanog* and maintained hESCs colony morphology temporarily under differentiation conditions. Our results indicate a direct link between histone modulation and post-transcriptional regulation in hESCs differentiation.

## Materials and Methods

### Cell Lines and Ethics Statement

The human ES cell line X-01 was a kind gift from Prof. Xiao, Zhejiang University, China [25].The study was approved by the Ethics Committee and Science Committee of the Second Military Medical University, China.

### Cell Culture

X-01 cells were cultured according to the WiCell Research Institute’s instructions. In brief, the cells were maintained on irradiation-inactivated CF-1 mouse embryonic fibroblast (MEF) [26], which were purchased from Applied StemCell (Menlo Park, CA, USA), in hESC culture medium on matrigel (BD Biosciences). hESCs were routinely passaged by enzymatic dissociation using Collagenase IV (Invitrogen) every 7 days and seeded in feeder-free cultures with conditioned medium on matrigel (BD Biosciences) for further investigations. Differentiation was induced using hESC medium BMP4 (50 ng/mL; Humanzyme) without bFGF for 9 days. Ellagic acid (100 µM) was added to the hESC medium for a site-specific inhibition of CARM1.

### Alkaline Phosphatase and Immunofluorescence Staining

hESCs were plated in 6-well plates (5×10^4^/well) or 12-well plates (2.5×10^4^/well) and fixed with 4% paraformaldehyde for 1–2 min. An alkaline phosphatase (AP) staining kit from SiDanSai Stem Cell Technology (Shanghai, China) was used according to the manufacturer’s protocol. Self-renewing colonies stain positive for AP, while differentiated colonies stain less or negative for AP. For immunofluorescence staining, hESCs were fixed with 4% paraformaldehyde and incubated with blocking solution (0.1%Triton X-100 and 5% normal goat serum in PBS) for 30 min at room temperature. To detect the expression of pluripotency markers, the cells were stained with anti-Oct4 (Abcam), anti-Nanog (Abcam) and anti-Sox2 (Abcam).

### RNA Isolation and Real-time PCR Analysis

Total RNA was extracted using Trizol (Invitrogen). For miRNA detection, reverse transcription was performed using microRNA specific stem-loop primers and real-time PCR was performed using TaqMan probes provided by TaqMan miRNA assays (Applied Biosystems). The *U6* RNA was used as an miRNA internal control. For mRNA detection, the first-strand cDNA was generated using the Reverse Transcription System Kit (Promega) with random primers and real-time PCR was performed using a standard SYBR-Green PCR kit protocol in a StepOne Plus system (Applied Biosystems). Marker genes for specific differentiated lineages were selected according to previous reports. *β-actin* was used as an endogenous control to normalize the amount of total mRNA in each sample. The primer sequences are presented in Table S1.

### siRNA and microRNA Synthesis

siRNAs specifically targeting *CARM1* (the sequences are depicted in Table S1) were synthesized by GenePharma (Shanghai, China). miR-181a, -181b, -181c and -181d mimics and control RNAs were synthesized by GenePharma (Shanghai, China). An siRNA that specifically targets and inhibits miR-181c was synthesized by GenePharma (Shanghai, China).

### Construction of Vectors

The complementary DNA encoding *CARM1* that lacks its 3′UTR was PCR-amplified from human genomic DNA using TaKaRa LA Taq (TaKaRa) and was subcloned into the pcDNA3.1-flag-vector (Invitrogen) to generate a *CARM1* RNAi-resistant expression vector. The 3′UTR of the *CARM1* mRNA was PCR-amplified by PrimeSTAR HS DNA Polymerase (TaKaRa) and subcloned into the pMIR-REPORT vector (Applied Biosystems) immediately downstream of the luciferase gene. The pMiR-REPORT-mut-*CARM1* 3′UTR constructs containing the *CARM1* 3′UTR with three point mutations in the seed sequence were synthesized with a QuikChange II Site-Directed Mutagenesis kit (Stratagene). All of the primer sequences are presented in Table S1.

### Transient Transfection

Transfections of plasmids and RNAs were performed using the Fugene HD reagent (Promega) and Lipofectamine 2000 (Invitrogen), respectively, according to the manufacturers’ instructions. The double-stranded microRNAs mimics, siRNAs and their respective negative control RNAs (GenePharma) were introduced into cells every 4 days at a final concentration of 50 nM.

### Luciferase Reporter Assay

5×10^3^ HEK293 cells were seeded into each well of 96-well plate and incubated overnight. Then, the cells were co-transfected with 80 ng of the pMIR-REPORT-*CARM1* 3′UTR plasmid or the pMIR-REPORT-mut-*CARM1* 3′UTR plasmid, 8 ng of the internal control pRL-TK-Renilla-luciferase plasmid and the indicated RNAs (final concentration, 50 nM). For hESCs, 200 ng of the pMIR-REPORT-*CARM1* 3′UTR plasmid or the pMIR-REPORT-mut-*CARM1* 3′UTR plasmid and 20 ng of the pRL-TK plasmid were transfected into the hESCs in a 24-well plate. After 48 h, the luciferase activities were measured using the Dual-Luciferase Reporter Assay System (Promega) according to the manufacturer’s instructions. The data were normalized by dividing the firefly luciferase activity by that of the Renilla luciferase.

### Western Blotting Analysis

Total cell lysates were prepared in a 1× sodium dodecyl sulfate buffer [27]. Identical quantities of proteins were separated by sodium dodecyl sulfate-polyacrylamide gel electrophoresis and transferred onto polyvinylidene fluoride membranes. The following antibodies were used for Western blotting: anti-CARM1 (Abcam), anti-histone H3R17di-me (Millipore), anti-Nanog (Abcam), and anti-Oct4 (Abcam). Anti-β-actin (Abcam) was used as endogenous control.

### Chromatin Immunoprecipitation Assay

Chromatin immunoprecipitation (ChIP) assays were performed using the EZ-Magna ChIP™ A/G Chromatin Immunoprecipitation Kit (Millipore). Chromatin was immunoprecipitated using anti-CARM1 (Abcam) and anti-histone H3R17di-me (Abcam). Anti-RNA polymerase (Millipore) was used as a positive control, and IgG (Santa Cruz) was used as a negative control. ChIP-derived DNA was quantified using real-time PCR with SYBR-Green incorporation (Applied Biosystems). Three pairs of primers were designed for the promoter region of each gene to detect the enriched genomic DNA fragments. The qPCR values were normalized to the values of a promoter region of GAPDH. The normalized input signal was defined as 1. The fold enrichment value is shown as the normalized ChIP signal divided by the normalized input signal. ChIP-PCR-derived DNA was also electrophoresed through 2% agarose gels. The primer sequences are presented in Table S1.

### Statistical Analysis

The data are presented as the mean ± SD. Comparison of the different parameters for protein, gene, miRNA, luciferase expression and AP-positive colony numbers and fold enrichments was determined by repeated measures analysis of variance (ANOVA). Significant differences were assigned using Dunnett’s post hoc test. Comparison of mature miR-181c expression was performed with an unpaired Student’s t test.

## Results

### *CARM1* is Down-regulated Post-transcriptionally during Human ESC Differentiation

**Figure 1:**
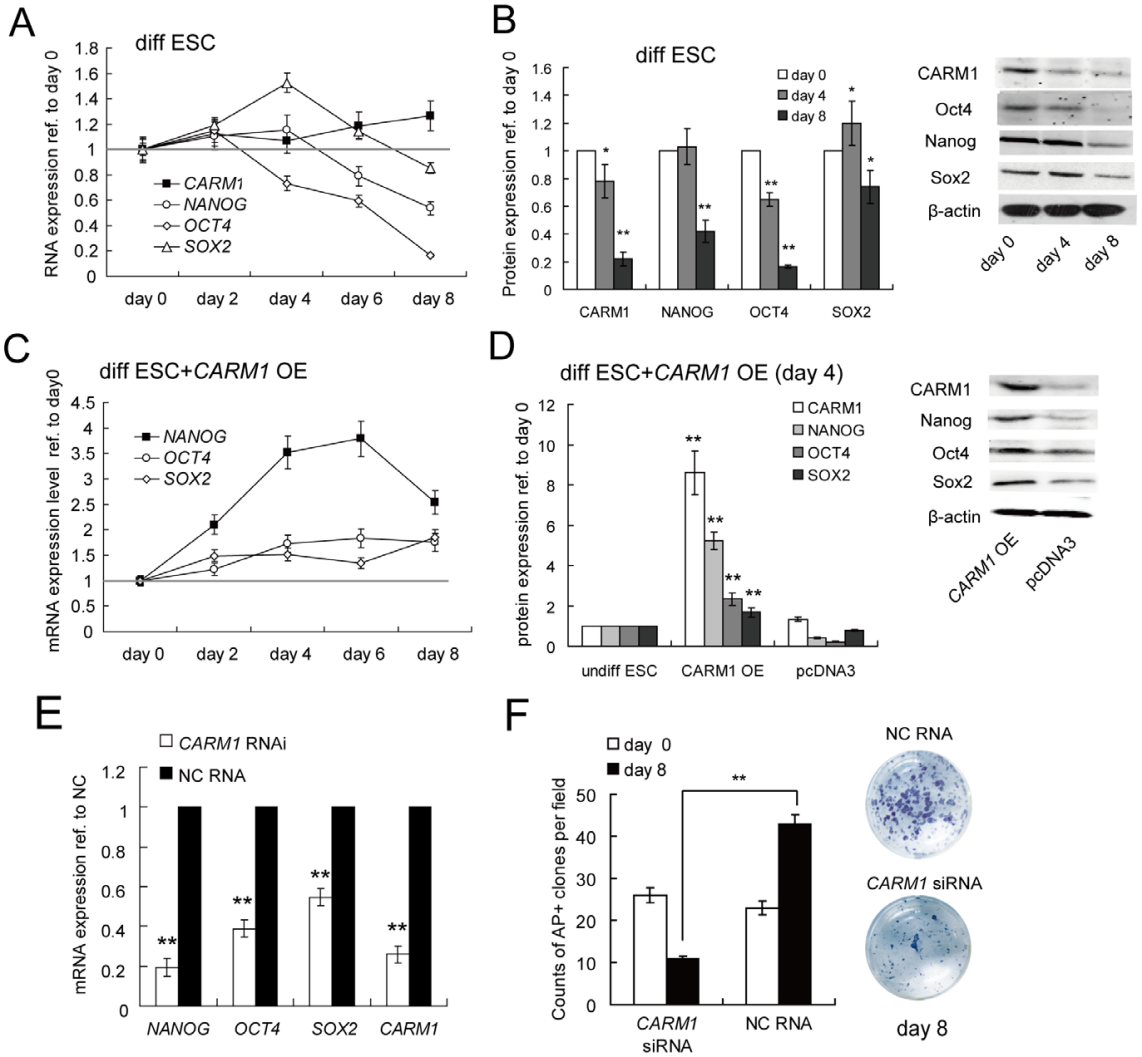
CARM1 is down-regulated post-transcriptionally during hESC differentiation. (A, B) The kinetic expression levels of *CARM1*, *Nanog*, *Sox2*, and *Oct4* in differentiated hESCs. Human ESCs differentiation was induced by the addition of BMP4 in the absence of bFGF. The expression levels of *CARM1*, *Nanog*, *Sox2*, and *Oct4* at the mRNA (A) and protein levels (B) were quantified by qRT-PCR and Western blotting, respectively. *, p<0.05; **, p<0.01. (C, D) Overexpression of 3′UTR-deficient *CARM1* rescued the down-regulation of key pluripotency factors during hESC differentiation. Differentiation of hESCs overexpressing 3′UTR-deficient *CARM1* was induced, and the expression levels of *CARM1*, *Nanog*, *Sox2*, and *Oct4* at the mRNA (C) and protein levels (D) were quantified by qRT-PCR and Western blotting, respectively. Blank pcDNA3 was used as a negative control. Samples were assayed in duplicate (n = 3) and normalized to endogenous *β-actin* expression. **, p<0.01. (E) Knockdown of *CARM1* in ESCs using siRNAs resulted in the down-regulation of *CARM1*, *Nanog, Sox2* and *Oct4* mRNA levels. *CARM1*-specific siRNAs were transfected every 4 days; total RNA was extracted from hESCs 8 days after the first transfection and quantified by quantitative real-time polymerase chain reaction (qRT-PCR). Mean values of the indicated transcript levels are plotted as percentages relative to those after transfection with negative control RNA (NC RNA). Samples were assayed in duplicate (n = 3) and normalized to endogenous *β-actin* expression. **, p<0.01. (F) The pluripotency of hESCs was examined by AP staining 8 days after siRNA transfection. NC RNA-transfected ESCs were used as controls. The counts of AP-positive clones per field were quantified in duplicate (n = 3), and the images for the representative plates are all shown. **, p<0.01.

To identify whether CARM1 is associated with the differentiation of hESCs, we analyzed the expression dynamics of *CARM1* during BMP4-induced hESC differentiation. We found that the transcript level of *CARM1* did not decline as expected. To the contrary, the *CARM1* expression levels were comparable to those of undifferentiated cells over the entire time course, whereas the expression levels of the pluripotency genes declined after 4 days of BMP4-induced differentiation (Fig. 1A). Interestingly, immunoblotting showed that the protein level of *CARM1* decreased gradually, consistent with the levels of pluripotency genes (Fig. 1B). This finding indicated that *CARM1* could be subjected to a post-transcriptional regulatory mechanism, e.g., miRNAs, which may inhibit protein translation without the degradation of its coding mRNA. To confirm the role of miRNAs in the regulation of the *CARM1* expression level during hESC differentiation, we generated an expression vector containing an miRNAs-resistant *CARM1* coding sequence that lacked the 3′UTR (pcDNA3.1-*CARM1*) and transfected it into undifferentiated hESCs. Interestingly, the expression dynamics of the pluripotency genes were different. *Oct4* and *Sox2* levels slightly increased whereas *Nanog* levels were up-regulated by 3.8-fold and 5.2-fold for mRNA and protein, respectively, after 6 days (Fig. 1C, D). Furthermore, the expression of the marker genes for specific differentiated lineages decreased on day 9 (Fig. S1), and the Nanog expression level was detected by immunofluorescence (Fig S4A). We found that *CARM1*-overexpressing cells maintained the normal morphologies for as long as 4 days in contrast to pcDNA3-overexpressing cells, which began to show characteristic differentiation morphologies after 1 day of BMP4-induced differentiation (Fig. S4B). AP staining after 4 days of differentiation is shown (Fig. S4B). It appears that hESCs in which *CARM1* was overexpressed 9-fold (Fig. 1D) do respond to differentiation signals, and the initiation of differentiation is considerably delayed for a period during which *Nanog* is expressed at a higher level. To confirm whether CARM1 plays a key role in modulating hESC differentiation, we knocked down *CARM1* transcript levels using *CARM1*-specific siRNAs in undifferentiated hESCs. We found that these siRNAs led to dramatic decreases in the expression levels of *CARM1* and pluripotency genes (Fig. 1E). hESCs in which *CARM1* was down-regulated formed less AP-positive colonies than wild-type cells (Fig. 1F), and the expression of the marker genes for specific differentiated lineages increased on day 8 after the knockdown of *CARM1* (Fig. S1B).

### miR-181 Family Members are Critical Regulators of CARM1 during hESC Differentiation

**Figure 2:**
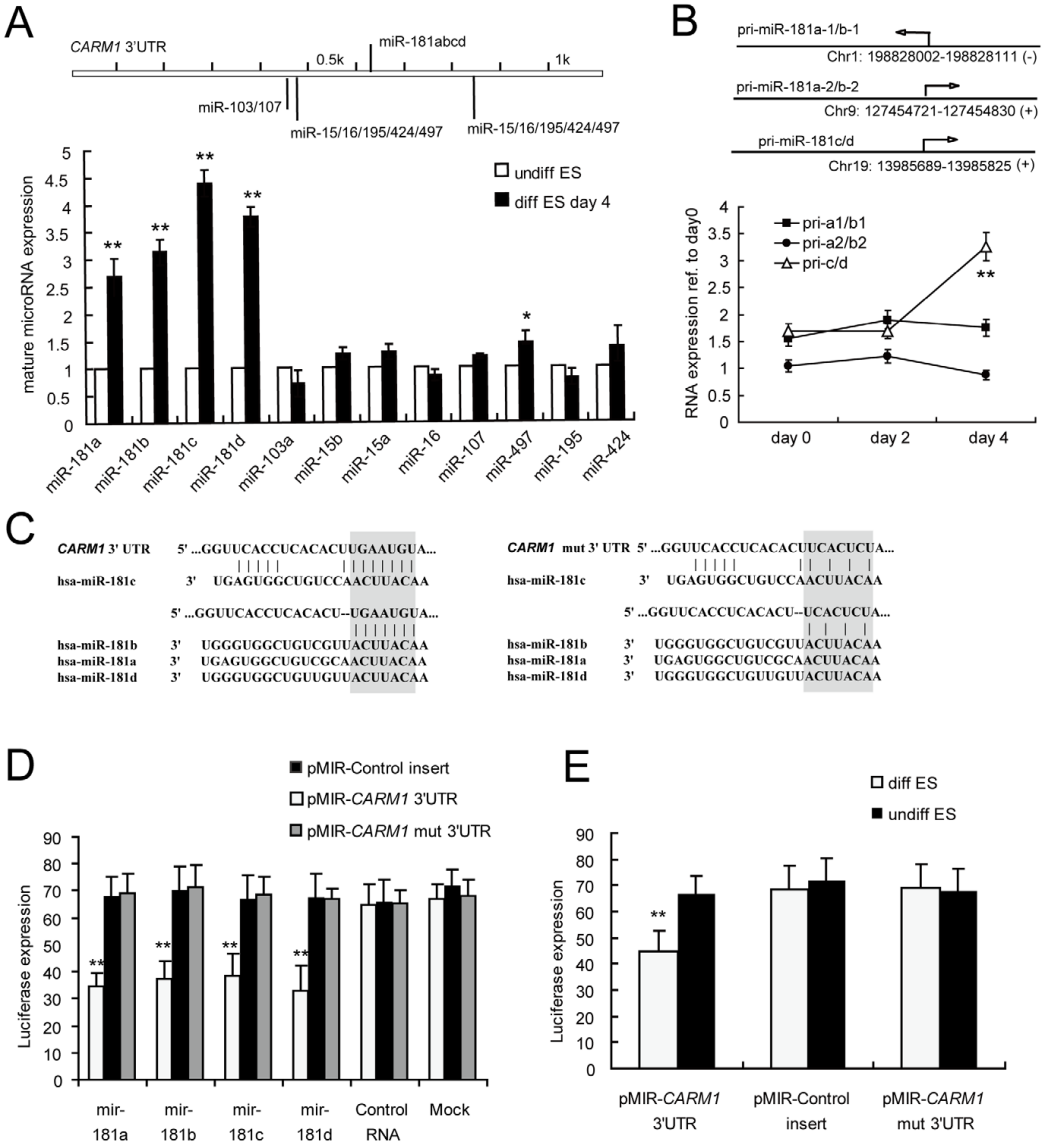
The miR-181 family directly regulates *CARM1* expression in hESC. (A) The expression levels of mature miRNAs predicted to target the *CARM1* 3′UTR were monitored in differentiated ESCs by qRT-PCR and normalized to endogenous *U6* expression. *, p<0.05; **, p<0.01. (B) The chromosome positions of the primary transcripts of miR-181 family members are shown, their expression levels in differentiated ESCs were determined by qRT-PCR and normalized to endogenous *β-actin* expression. **, p<0.01. (C) The predicted consequential pairing of miRNAs and their target regions in the wild-type *CARM1* 3′UTR or the mutant (mut) *CARM1* 3′UTR are shown. (D) The HEK293 cells were co-transfected with the firefly-luciferase-expressing vector pMIR-REPORT containing the wild-type *CARM1* 3′UTR, the mut *CARM1* 3′UTR or the control insert as well as the internal control renilla-luciferase-expressing vector pRL-TK and the indicated RNAs. After 48 h, the luciferase activities were measured. The data were normalized by dividing firefly luciferase activity with that of Renilla luciferase, **, p<0.01. (D) Luciferase activities were measured in human ESCs co-transfected with the pMIR-REPORT plasmid containing wild-type *CARM1* 3′UTR, mut *CARM1* 3′UTR or control insert and the internal control, pRL-TK. All the samples were assayed in duplicate (n = 3). **, p<0.01.

Considering that the CARM1 protein expression level was greatly decreased whereas mRNA level remain unchanged during hESCs differentiation, it was important to consider the post-transcriptional mechanisms responsible for the down-regulation of *CARM1* during differentiation. We used the bioinformatics tool TargetScan to search for miRNAs that target the *CARM1* 3′UTR. More than 25 candidate miRNAs were predicted to target the *CARM1* 3′UTR, and the 12 miRNAs with the highest context scores (Table S2) were assayed using qRT-PCR in undifferentiated hESCs and differentiated hESCs. The mature transcripts of the 4 members of the miR-181 family were all found to be significantly increased in differentiated hESCs, and miR-181c had the highest expression level (Fig. 2A). We also found that the expression levels of the miR-181c/d primary transcripts (pri-181c/d) were notably elevated after differentiation in comparison to the primary transcripts of miR-181a and miR-181b (pri-181a1/b1 and pri-181a2/b2) (Fig. 2B). To investigate whether CARM1 can be directly targeted by miR-181, we engineered luciferase reporters that have either the wild-type 3′UTR of CARM1, or a mutant 3′UTR with three point mutations in the target sites as a negative control (Fig. 2C). The luciferase reporters were co-transfected with miR-181a/b/c/d mimics into HEK293 cells. We found that the mimics of miR-181a/b/c/d significantly reduced the luciferase activities of the wild-type CARM1 reporters in comparison to the negative control. By contrast, the expression of mutant reporters was not repressed by miR-181 (Fig. 2D). To study the role of endogenous miR-181 in repressing the *CARM1* 3′UTR reporter in differentiated hESCs, we co-transfected the wild-type 3′UTR luciferase reporter and the negative control luciferase into differentiated hESCs. At 24 hours after transfection, we found significant repression of the wild-type 3′UTR luciferase reporter activity of *CARM1* in comparison to the negative control (Fig. 2E). Taken together, these results show that miR-181 directly regulates *CARM1* by targeting its 3′UTR and that miR-181c may play a prominent role among the 4 members during hESC differentiation.

### Enforced Expression of miR-181c Induced hESC Differentiation by Targeting *CARM1*

**Figure 3:**
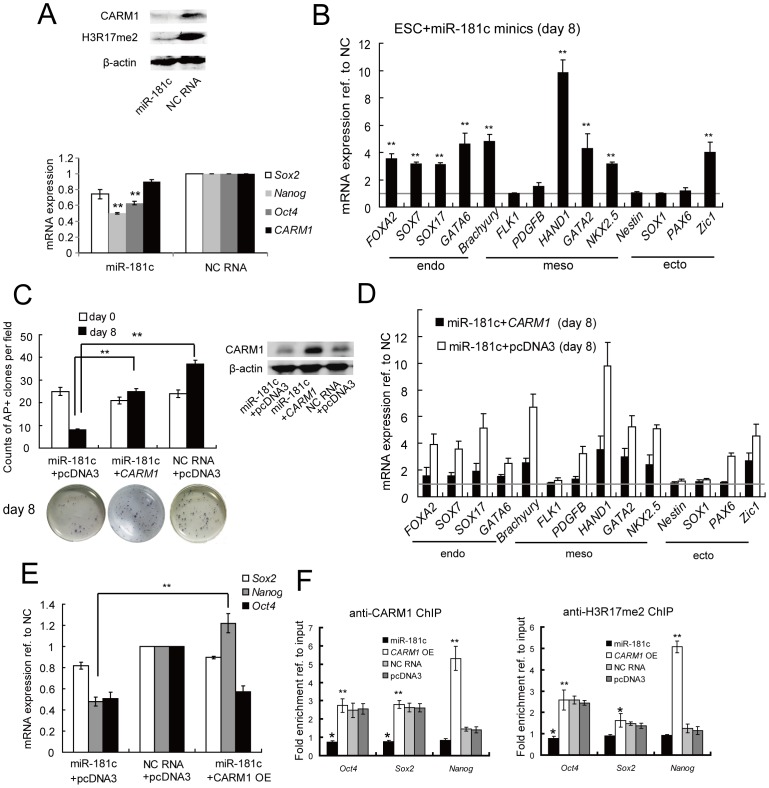
miR-181c leads to hESC differentiation through negative regulation of *CARM1* and H3R17 methylation. (A) Overexpression of miR-181c down-regulated *CARM1* expression in comparison to negative control (NC) RNA-transfected ESCs at both the mRNA and protein levels, as determined by qRT-PCR and Western blotting, respectively. H3R17 methylation level and *Oct4*, *Nanog* and *Sox2* mRNA expression were also monitored. Samples were assayed in duplicate (n = 3) and normalized to endogenous *β-actin* expression. **, p<0.01. (B) Expression of a subset of differentiation genes in ESCs that were transfected with miR-181c or NC RNA or were not transfected was monitored by qRT-PCR. Mean levels (after 3 days) of expression are shown relative to the NC RNA-transfected sample (shown as one fold) and normalized to *β-actin* expression levels. **, p<0.01. (C, D, E) miR-181c mimics were transfected into ESCs with the 3′UTR-deficient-*CARM1*-expressing plasmid (*CARM1* OE) or the control plasmid (pcDNA3). ESCs co-transfected with NC RNA and pcDNA3 were used as controls. (C) Pluripotency was examined by AP staining 8 days after transfection. The images for the whole plate or representative clones are shown. Scale bar: 500 µm. CARM1 protein expression is also shown. **, p<0.01. (D) Expression of a subset of differentiation genes in ESCs transfected with miR-181c+pcDNA3 or miR-181c+*CARM1* or NC RNA was monitored by qRT-PCR. Mean levels (after 3 days) of expression are shown relative to the NC RNA-transfected sample (shown as one fold) and normalized to *β-actin* expression levels. (E) *Oct4*, *Nanog* and *Sox2* mRNA expression was also monitored by qRT-PCR. Samples were assayed in duplicate (n = 3) and normalized to endogenous *β-actin* expression. **, p<0.01. (F) ChIP analysis of hESCs overexpressing *CARM1* or miR-181c. ChIP was performed on sonicated chromatin using anti-CARM1 or anti-H3R17-di-me antibodies. Immunoprecipitated DNA was analyzed by qRT-PCR with primers targeting the promoter regions of the investigated gene. The fold enrichment value is shown as the normalized ChIP signal divided by the normalized input signal. Cells transfected with NC RNA or pcDNA3 were used as negative controls. The results of the electrophoretic analysis are also shown in Fig. S3A. *, p<0.05; **, p<0.01.

We selectively transfected miR-181c mimics in undifferentiated hESCs to study the effect of miR-181 on hESCs differentiation. Enforced expression of miR-181c led to a clear down-regulation of *CARM1* expression at the protein level (Figure 3A). At the same time, the expression of *Oct4*, *Sox2*, and *Nanog* decreased (Figure 3A), whereas the expression of the marker genes for specific differentiated lineages increased (Figure 3B). Additionally, similarly to knock down *CARM1* transcript levels using siRNAs, hESC differentiation was also marked by the loss of AP-positive colonies (Figure 3C).

Furthermore, to understand whether miR-181c overexpression induces differentiation mainly by regulating *CARM1* expression, we co-transfected miR-181c mimics and a *CARM1* miRNA-resistant expression vector into undifferentiated hESCs and found that co-expression of *CARM1* and miR-181c maintained the number of AP-positive colonies independent of the expression of miR-181c (Figure 3C), CARM1 protein expression is also shown (Figure 3C). The expression of *Nanog* and the marker genes for specific differentiated lineages was restored in comparison to miR-181c -overexpressing cells (Figure 3D, 3E). All the results indicated that *CARM1* down-regulation may greatly contribute to the miR-181-meidated hESC differentiation.

### Enforced Expression of miR-181c Suppresses CARM1-mediated *Nanog* Transcription

In previous reports, histone H3 methylation on R17 has been identified as the main substrate of CARM1 in mouse ESCs. In our results from miR-181c-transfected hESCs, we also found that the global level of histone H3 methylation on R17 was also significantly decreased (Figure 3A). To further reveal the mechanism of CARM1-mediated gene regulation, we then assessed whether the CARM1-mediated histone H3 methylation contributed to the regulation of pluripotency genes *Oct4*, *Sox2* and *Nanog* after miR-181c transfection.

Firstly, to identify the role of histone H3 methylation at R17 catalyzed by CARM1 during hESC differentiation, a histone H3R17 methylation-specific inhibitor, ellagic acid [28], was employed. This site-specific inhibitor treatment caused a significant reduction of the *Nanog* expression level compared to undifferentiated hESCs (Fig. S2A). Differentiation was also marked by the up-regulation of the marker genes for specific differentiated lineages after inhibitor treatment for 5 days (Fig. S2B).

To further investigate whether CARM1 directly targets the pluripotency genes *Oct4*, *Sox2* and *Nanog*, we performed ChIP analysis on wild-type hESCs, miR-181c- overexpressing hESCs and *CARM1*-overexpressing hESCs as well as hESCs transfected with negative control (NC) RNA or pcDNA3. ChIP-derived DNA amplified by qRT-PCR was electrophoresed through 2% agarose gels (Fig. S3A), and the quantitative data are shown in Fig. 3F. We found that CARM1 and histone H3R17 di-me were significantly enriched at the promoters of *Oct4* and *Sox2* in both NC RNA/pcDNA3-overexpressing hESCs and *CARM1*-overexpressing hESCs, whereas this enrichment was clearly decreased in miR-181c-overexpressing hESCs. Unexpectedly, the Nanog promoter did not show any detectable enrichment in NC RNA/pcDNA3-overexpressing hESCs or miR-181c-overexpressing hESCs, but we found that CARM1 and histone H3R17 di-me were significantly enriched at the *Nanog* promoter in *CARM1*-overexpressing hESCs (Fig. 3F). These results suggest that CARM1 directly catalyzes histone H3 arginine methylation of the promoters of *Oct4* and *Sox2* and that ectopic expression of CARM1 leads to its recruitment to the *Nanog* promoter.

### Suppression of miR-181c Impedes hESC Differentiation through the CARM1-related Pathway

**Figure 4:**
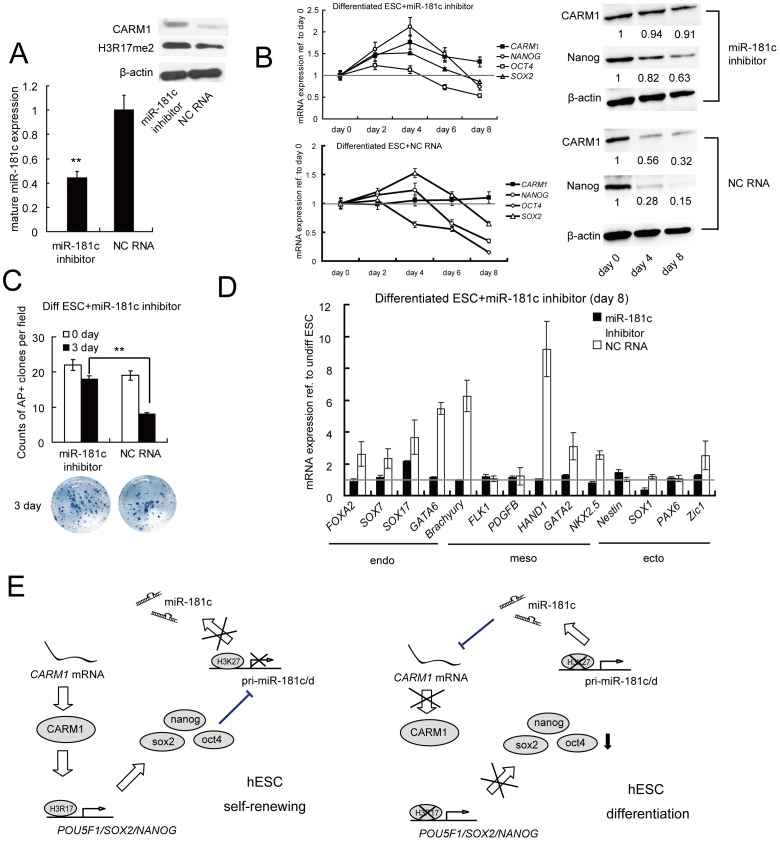
The miR-181c inhibitor suppresses hESC differentiation. (A) Enforced expression of the miR-181c inhibitor down-regulated mature miR-181c levels relative to negative control (NC) RNA-transfected ESCs, as shown by qRT-PCR. **, p<0.01. Western blotting detected that CARM1 and H3R17me2 protein levels were clearly decreased. (B, C, D) The effect of miR-181c inhibition on differentiated hESCs. Human ESCs were transfected with miR-181c inhibitor or NC RNA and then induced to differentiate by the addition of BMP4 in the absence of bFGF. (B) The expression of *CARM1*, *Nanog*, *Sox2*, and *Oct4* at the mRNA level was quantified by qRT-PCR, CARM1 and Nanog protein expression levels were also quantified by Western blotting. (C) Pluripotency was examined by AP staining 3 days after transfection. The counts of AP-positive clones and the images of the representative plates are shown. Samples were assayed in duplicate (n = 3). **, p<0.01. (D) Expression of a subset of differentiation-associated genes in ESCs transfected with miR-181c inhibitor or NC RNA were monitored by qRT-PCR and normalized to *β-actin* expression levels. Mean levels (after 8 days) expressed relative to undifferentiated hESCs (shown as one fold) are shown. (E) Model for the miR-181/CARM1/core-pluripotency-factors regulatory loop in the modulation of hESC pluripotency. Pluripotency is maintained in ESCs in part by histone H3 arginine methylation by CARM1 at the *Oct4*, *Nanog* and *Sox2* promoters. The core pluripotency factors also recruit H3K27 methylases to the miR-181c promoter to inhibit its expression. In differentiated hESCs, H3K27 methylation is inhibited due to the reduction of core pluripotency factors, and miR-181 family members are subsequently induced and down-regulate CARM1 activity. H3R17me2 production is eventually stopped, which aggravates the decrease in expression of core pluripotency factors as well as the loss of pluripotency.

To further assess the effect of miR-181c on hESC differentiation, we knocked down miR-181c using a specific inhibitor and found that CARM1 and H3R17me2 protein expression levels were clearly down-regulated in comparison to hESCs treated with NC RNA (Figure 4A). *CARM1* expression levels were significantly increased for mRNA and protein in comparison to hESCs treated with NC RNA after 4 and 8 days (Figure 4B). The mRNA expression levels of *Nanog* were up-regulated by 2.2-fold and declined gradually after 4 days. The protein levels were significantly increased in comparison to hESCs treated with NC RNA after 4 and 8 days. *Oct4* and *Sox2* mRNA levels slightly increased before they declined to levels comparable to those of the controls after 2 days (Figure 4B). Suppression of miR-181c led to a comparable number of AP-positive colonies relative to wild-type cells (Figure 4C), and the expression of most of the marker genes for specific differentiated lineages was restored to wild-type levels (Figure 4D). ChIP analysis detected that CARM1 and histone H3R17 di-me were significantly enriched at the *Nanog* promoter in antago-miR-181c-overexpressing hESCs (Fig. S3B). ChIP-derived DNA amplified by qRT-PCR was electrophoresed through 2% agarose gels (Fig. S3C). The elevated expression level of Nanog relative to that in NC RNA-treated cells was detected by immunofluorescence (Fig. S4C).

## Discussion

Histone modifications are pivotal for the transmission of cell fate information during human ESC self-renewal and differentiation [29], [30]. Activating (H3K4me3) and repressive (H3K27me3) histone lysine methylations are known to be associated with the transcription and repression of gene expression, respectively [10], [31]. The histone modulators that regulate these modifications, including the Trithorax and Polycomb complexes, are also known to be important for the regulation of gene expression in the plastic chromatin of hESCs [32], [33], [34]. However, in current studies involving hESCs, few investigators consider the importance of H3 arginine methylation, another histone chemical modification that greatly contributes to mouse embryo development and the maintenance of mouse ES cell pluripotency [13], [14]. In this study, we demonstrate that CARM1, one of the key regulators of H3 arginine methylation, also contributes to maintaining the pluripotency of hESCs through the loss-of-function and gain-of-function studies. Our results suggest that H3 arginine methylation also plays a key role in the regulation of hESC self-renewal and differentiation which contributes to the current data regarding mouse ESCs.

Our results also suggest that the miR-181/CARM1/core-pluripotency-factors regulatory loop may be a novel model pathway involved in the modulation of hESC pluripotency (Figure 4E). Pluripotency is maintained in ESCs in part as a result of the arginine methylation of histone H3 by CARM1 at the *Oct4*, *Nanog* and *Sox2* promoters. As reported in previous studies [35], the core pluripotency factors co-occupied the promoters of pri-miR-181c/d with Polycomb group proteins, which increased local H3 lysine 27 (H3K27) methylation and inhibited miR-181c expression. In differentiated hESCs, H3K27 methylation is inhibited because of the reduction of core pluripotency factors, and miR-181 family members are consequently significantly induced and down-regulate CARM1 activity. H3R17me2 production is eventually stopped, which aggravates the decrease in the expression of core pluripotency factors as well as the loss of pluripotency.

Previous studies have identified that many crucial transcription factors, such as Oct4, Nanog and Sox2, form transcriptional circuitry and participate in auto- and cross-regulatory interactions to increase their own expression and that of other self-renewal-associated genes while repressing genes that promote differentiation [36], [37]. In our study, *CARM1* knockdown in hESCs impaired the expression of these transcription factors and promoted differentiation. We also found that CARM1 and histone H3R17 di-me were significantly enriched at the promoters of *Oct4* and *Sox2*, which indicated that CARM1 contributes to the expression of these two genes in undifferentiated ESCs. By contrast, the overexpression of *CARM1* induced an elevation of *Nanog* expression in hESCs, while the enrichment of CARM1 significantly increased at the *Nanog* promoter during *CARM1* overexpression. These results are similar to those of previous studies of mouse blastomeres and mESCs, which showed that the overexpression of *CARM1* led to an early and marked up-regulation of *Nanog* [13], [14] and thus suggested that the CARM1 protein is recruited to the *Nanog* promoter to activate its transcription by catalyzing histone H3 arginine methylation. Recent studies indicated that the nuclear receptor coactivator 3 (Ncoa3) binds to the *Nanog* promoter and recruits CARM1 to activate *Nanog* expression [38], the activity and stability of Ncoa3 are regulated by CARM1-dependent methylation simultaneously [39]. Thus, we hypothesize that Ncoa3 and CARM1, co-occupying at the *Nanog* promoter, may form a positive feedback loop regulating its expression. In addition, it is known that histone deacetylase Sirtuin 1 (SIRT1) regulates Nanog expression in mouse ESC and IPS by regulating p53 expression [40], [41]. A recent study reported that SIRT1 mRNA is stabilized by CARM1-dependent methylated RNA-binding protein (HuR) in hESC, CARM1 knockdown resulted in the loss of methyl-HuR and a marked decrease in SIRT1 [42]. Given the down-regulation of *Nanog* expression after histone H3R17 methylation-specific inhibitor treatment, it can be concluded that CARM1 plays an active role in resisting differentiation by elevating *Nanog* expression not only by its catalytic activity but also as a transcriptional co-activator in hESCs.

MicroRNA is another important epigenetic regulator in human ESCs [43], [44], [45]. Considering that the *CARM1* mRNA level did not decline even though its protein level was significantly decreased during hESC differentiation, we suspected that microRNA-mediated post-transcriptional mechanisms might contribute to the downregulation of *CARM1* during ESC differentiation. In this context, we scanned for microRNAs that target *CARM1* and finally identified the miR-181 family as the critical regulator of *CARM1* expression. The depletion of CARM1 and histone H3R17 di-me at the promoters of pluripotency genes in miR-181c-overexpressing hESCs reveals that enforced expression of miR-181c induces differentiation independent of BMP4 by targeting *CARM1*. By contrast, the suppression of miR-181c promotes the recruitment of endogenous CARM1 to the promoters of pluripotency genes, especially *Nanog*, to impede differentiation. This blockade implicated miR-181c as a prominent regulator of differentiation. Although the miR-181 family also regulates many target genes [21], [46], [47], [48], [49], it is important to highlight that in mouse ESCs, the miR-181 family regulates another histone modulator, Cbx7, which plays a critical role in maintaining ESC pluripotency [50]. This finding suggests that the miR-181 family may also promote differentiation by affecting histone modulation in mESCs. Considering that the sites of the *CARM1* 3′UTR that are targeted by miR-181 family members are conserved in mammals, we suppose that the interaction between miR-181 and CARM1 is conserved in mESCs. Meanwhile, we found that *CARM1* overexpression greatly rescued the effects of miR-181c on promoting ESC differentiation. Thus, we suggest that *CARM1* is one of the key target genes of the miR-181 family during the progression of ESCs differentiation.

### Conclusion

In summary, the work presented here elucidates a direct link between histone modulation and post-transcriptional regulation and reveals important insights into the role of CARM1/H3 arginine methylation in the differentiation of hESC. CARM1 achieves its effects at least in part through histone H3 arginine methylation at the promoters of key pluripotency genes. Our work suggests that downstream targets of the miR-181 family include epigenetic factors that reconfigure the H3 arginine methylation signature during the process of hESC differentiation. Our findings therefore offer a unique perspective for understanding the mechanisms of the differentiation of hESCs. Future studies will explore how the expression of the miR-181 family is regulated in ESC differentiation and whether other transcriptional factors are associated with CARM1.

## Supporting Information

Figure S1 **Expression of differentiation marker genes in** ***CARM1*** **-overexpressing and** ***CARM1*** **knock down hESCs.** (A) hESCs overexpressing miRNA-resistant-*CARM1* were induced to differentiate by the addition of BMP4 in the absence of bFGF. The blank pcDNA3 vector was used as a negative controls. Expression of a subset of differentiation marker genes in hESCs was monitored by quantitative real-time polymerase chain reaction (qRT-PCR) and normalized to *β-actin* expression levels. Mean expression levels (after 9 days) of each gene are shown as fold changes relative to the expression levels in undifferentiated hESCs (at day 0, shown as the gray line). (B) Expression of a subset of differentiation markers in ESCs transfected with *CARM1* siRNAs was monitored by qRT-PCR and normalized to *β-actin* expression levels. Mean levels (after 8 days) are expressed relative to the NC RNA (day 8, shown as the gray line). **, p<0.01.
(TIF)

Figure S2 **Specific inhibition of CARM1-mediated histone arginine methylation impaired hESC pluripotency.** Specific inhibition of CARM1-mediated histone arginine methylation in hESCs was performed with 100 µM ellagic acid. The expression levels of *CARM1*, *Nanog*, *Sox2*, and *Oct4* at the mRNA levels (A) and the mRNA expression levels of a subset of differentiation markers (B) were quantified by quantitative real-time polymerase chain reaction (qRT-PCR) 5 days after ellagic acid treatment, and the mean values of the indicated transcript levels are shown as fold changes relative to the expression levels in undifferentiated hESCs. Samples were assayed in duplicate (n = 3) and normalized to endogenous *β-actin* expression. **, p<0.01.
(TIF)

Figure S3 **ChIP analysis of hESCs after miR-181c,** ***CARM1*** **and miR-181c inhibitor overexpression.** ChIP was performed on sonicated chromatin from wild-type ES cells, *CARM1*- overexpressing cells, miR-181c-overexpressing cells and cells treated with the miR-181c inhibitor using anti-CARM1, anti-histone H3R17di-me antibodies, anti-RNA Polymerase antibodies and control IgG antibodies. Cells transfected with NC RNA or pcDNA3 were used as negative controls. The immunoprecipitated DNA was analyzed with semi-quantitative PCR, and the results of electrophoretic analysis are shown (A, C). CARM1 protein expression was detected by Western Blotting (A). Immunoprecipitated DNA was also analyzed by qRT-PCR as shown (B). *, p<0.05; **, p<0.01.
(TIF)

Figure S4 **Changes of pluripotency and cell morphology in response to overexpression of** ***CARM1*** **and inhibition of miR-181c upon induction of hESC differentiation.** *CARM1*-overexpressing hESCs still expressed pluripotency markers after 8 days of BMP4-induced differentiation (A) (data for Oct4 and Sox2 not shown). *CARM1*-overexpressing hESCs maintained normal morphologies for as long as 4 days after the induction of differentiation (B),and their pluripotency was indicated by AP-positive colonies observed on day 4 (B). Scale bar: 200 µm in (A), 500 µm in (B). hESCs treated with miR-181c inhibitor expressed Nanog after 8 days of differentiation (C). Scale bar: 200 µm.
(TIF)

Table S1 **Oligonucleotide Sequences used in this study.**
(DOC)

Table S2 **Predicted miRNAs target** ***CARM1*** **3′UTR.**
(DOC)
