# Supplementary figures and images for: MicroRNA-181 Regulates CARM1 and Histone Aginine Methylation to Promote Differentiation of Human Embryonic Stem Cells
Source: PLoS One. 2013 Jan 3;8(1):e53146. doi: 10.1371/journal.pone.0053146 (PMC3536801; doi:10.1371/journal.pone.0053146)

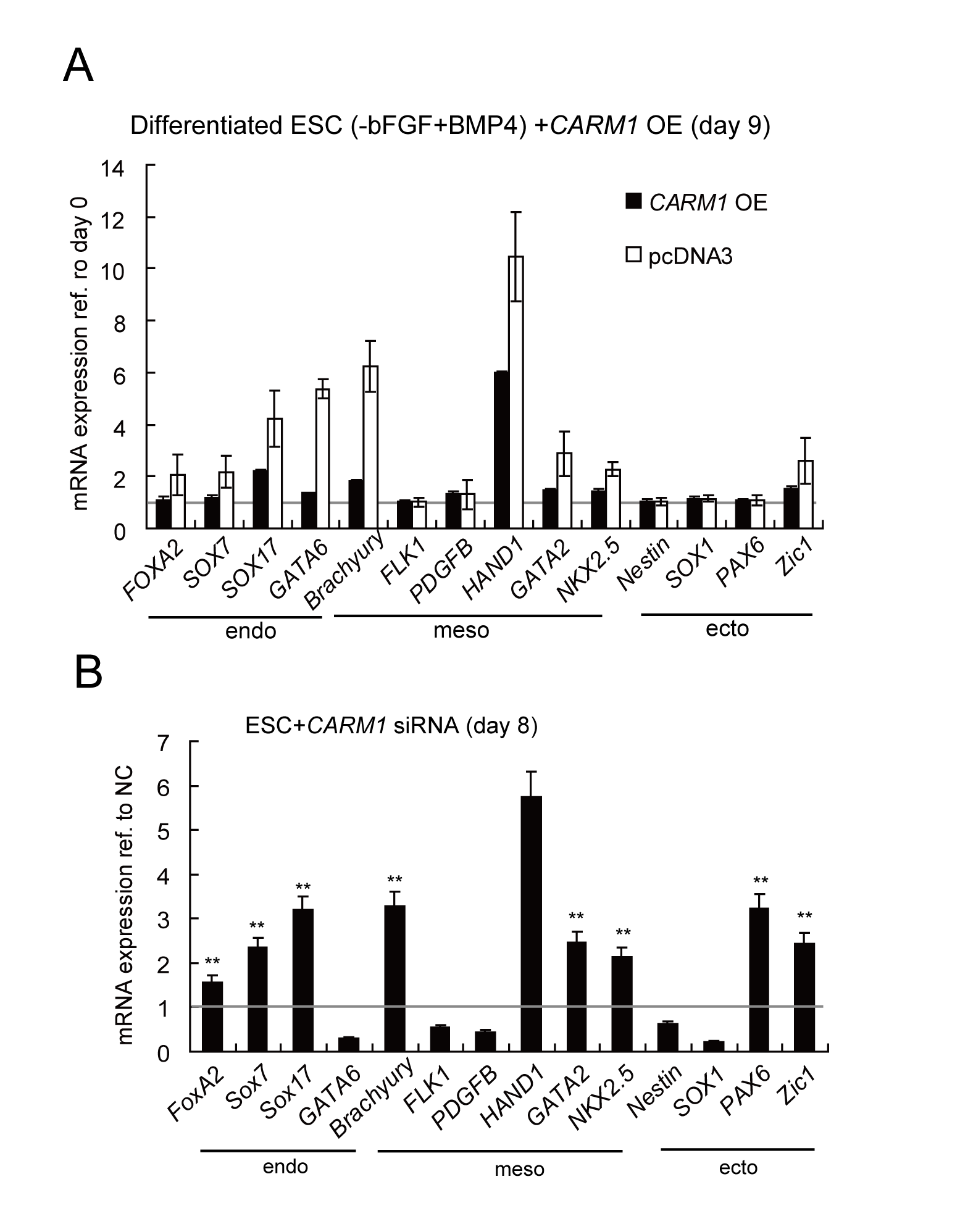

Supplement: Figure S1 — Expression of differentiation marker genes in CARM1 -overexpressing and CARM1 knock down hESCs. (A) hESCs overexpressing miRNA-resistant-CARM1 were induced to differentiate by the addition of BMP4 in the absence of bFGF. The blank pcDNA3 vector was used as a negative controls. Expression of a subset of differentiation marker genes in hESCs was monitored by quantitative real-time polymerase chain reaction (qRT-PCR) and normalized to β-actin expression levels. Mean expression levels (after 9 days) of each gene are shown as fold changes relative to the expression levels in undifferentiated hESCs (at day 0, shown as the gray line). (B) Expression of a subset of differentiation markers in ESCs transfected with CARM1 siRNAs was monitored by qRT-PCR and normalized to β-actin expression levels. Mean levels (after 8 days) are expressed relative to the NC RNA (day 8, shown as the gray line). **, p<0.01. (TIF) [file pone.0053146.s001.tif]

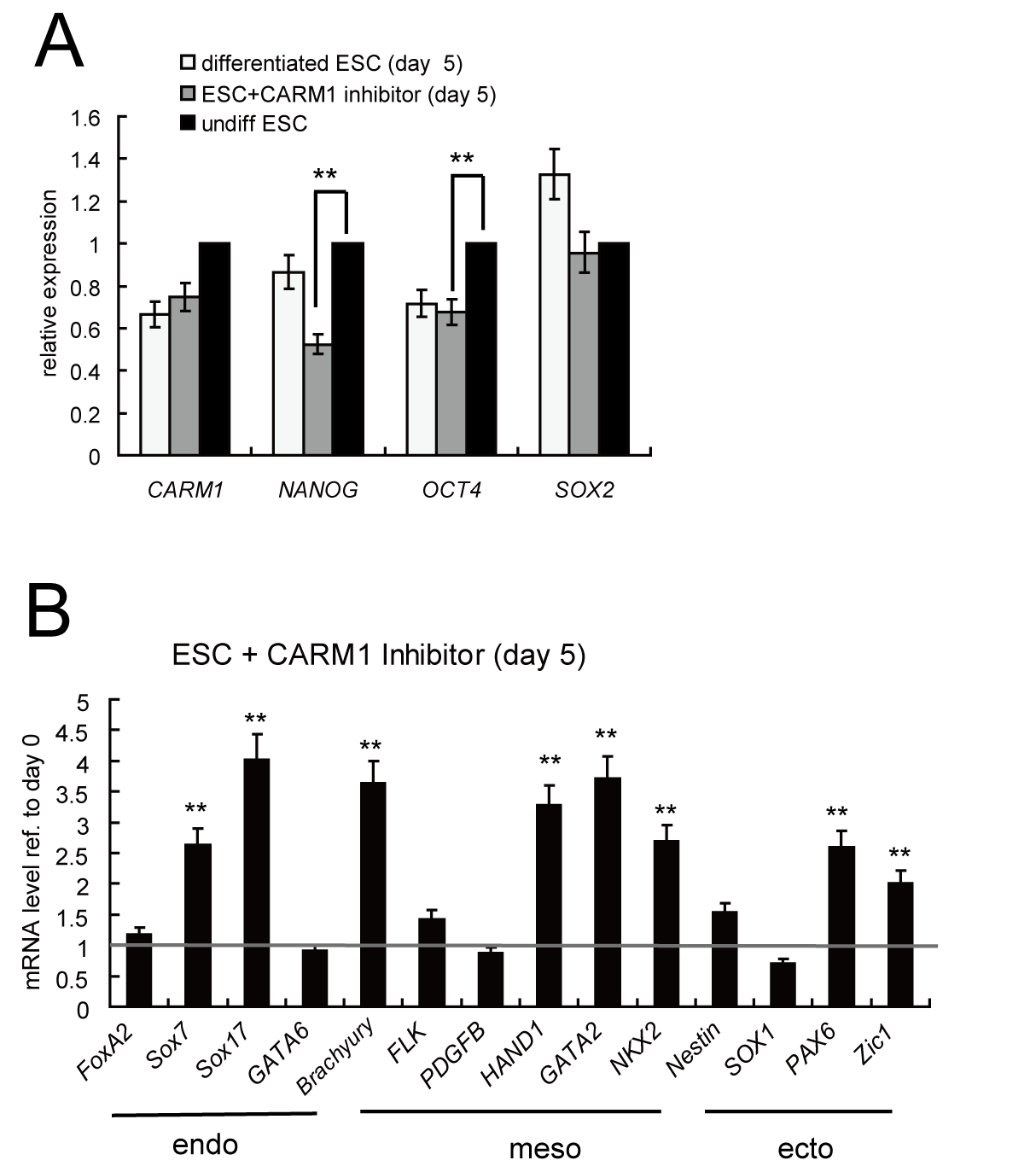

Supplement: Figure S2 — Specific inhibition of CARM1-mediated histone arginine methylation impaired hESC pluripotency. Specific inhibition of CARM1-mediated histone arginine methylation in hESCs was performed with 100 µM ellagic acid. The expression levels of CARM1, Nanog, Sox2, and Oct4 at the mRNA levels (A) and the mRNA expression levels of a subset of differentiation markers (B) were quantified by quantitative real-time polymerase chain reaction (qRT-PCR) 5 days after ellagic acid treatment, and the mean values of the indicated transcript levels are shown as fold changes relative to the expression levels in undifferentiated hESCs. Samples were assayed in duplicate (n = 3) and normalized to endogenous β-actin expression. **, p<0.01. (TIF) [file pone.0053146.s002.tif]

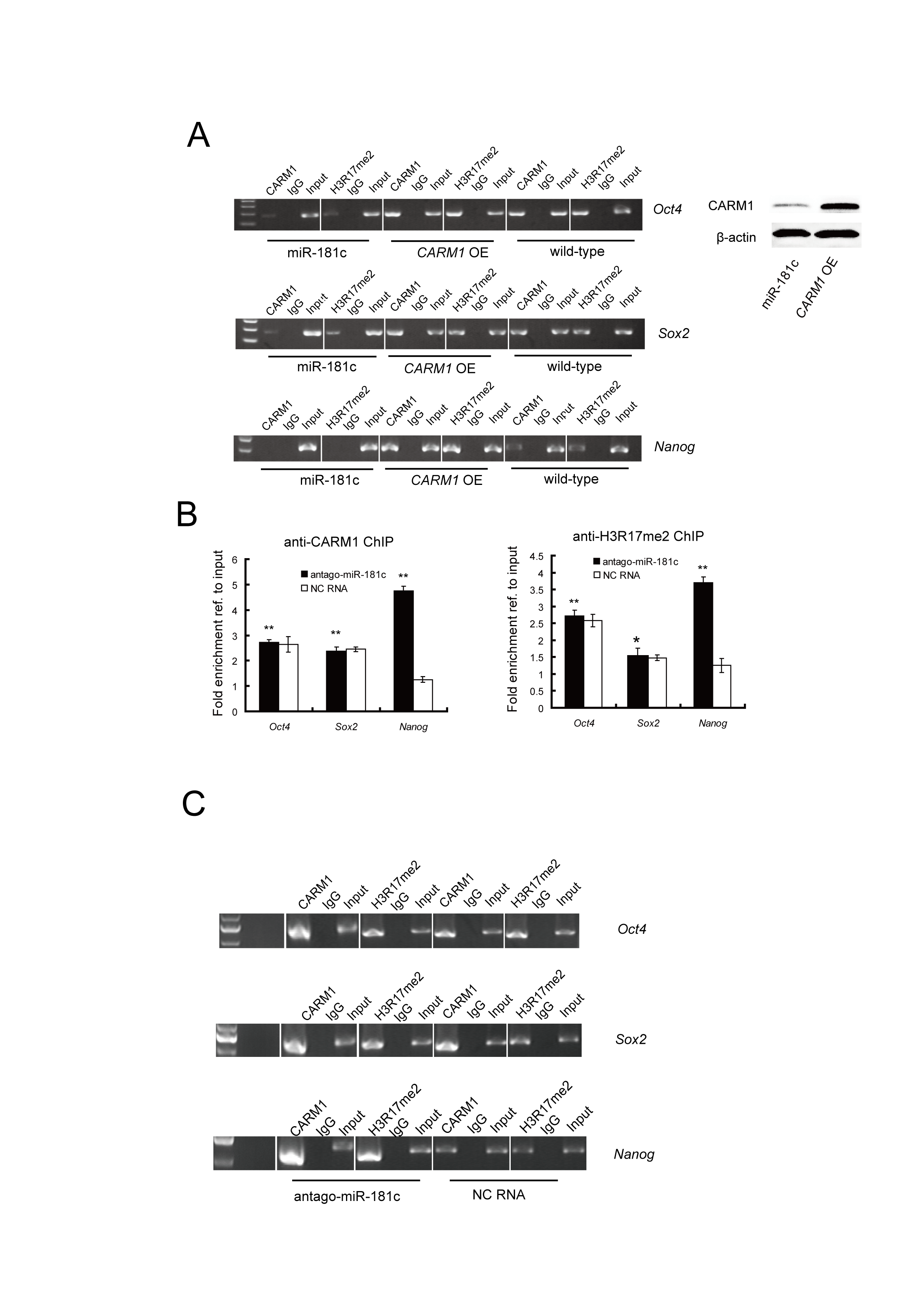

Supplement: Figure S3 — ChIP analysis of hESCs after miR-181c, CARM1 and miR-181c inhibitor overexpression. ChIP was performed on sonicated chromatin from wild-type ES cells, CARM1- overexpressing cells, miR-181c-overexpressing cells and cells treated with the miR-181c inhibitor using anti-CARM1, anti-histone H3R17di-me antibodies, anti-RNA Polymerase antibodies and control IgG antibodies. Cells transfected with NC RNA or pcDNA3 were used as negative controls. The immunoprecipitated DNA was analyzed with semi-quantitative PCR, and the results of electrophoretic analysis are shown (A, C). CARM1 protein expression was detected by Western Blotting (A). Immunoprecipitated DNA was also analyzed by qRT-PCR as shown (B). *, p<0.05; **, p<0.01. (TIF) [file pone.0053146.s003.tif]

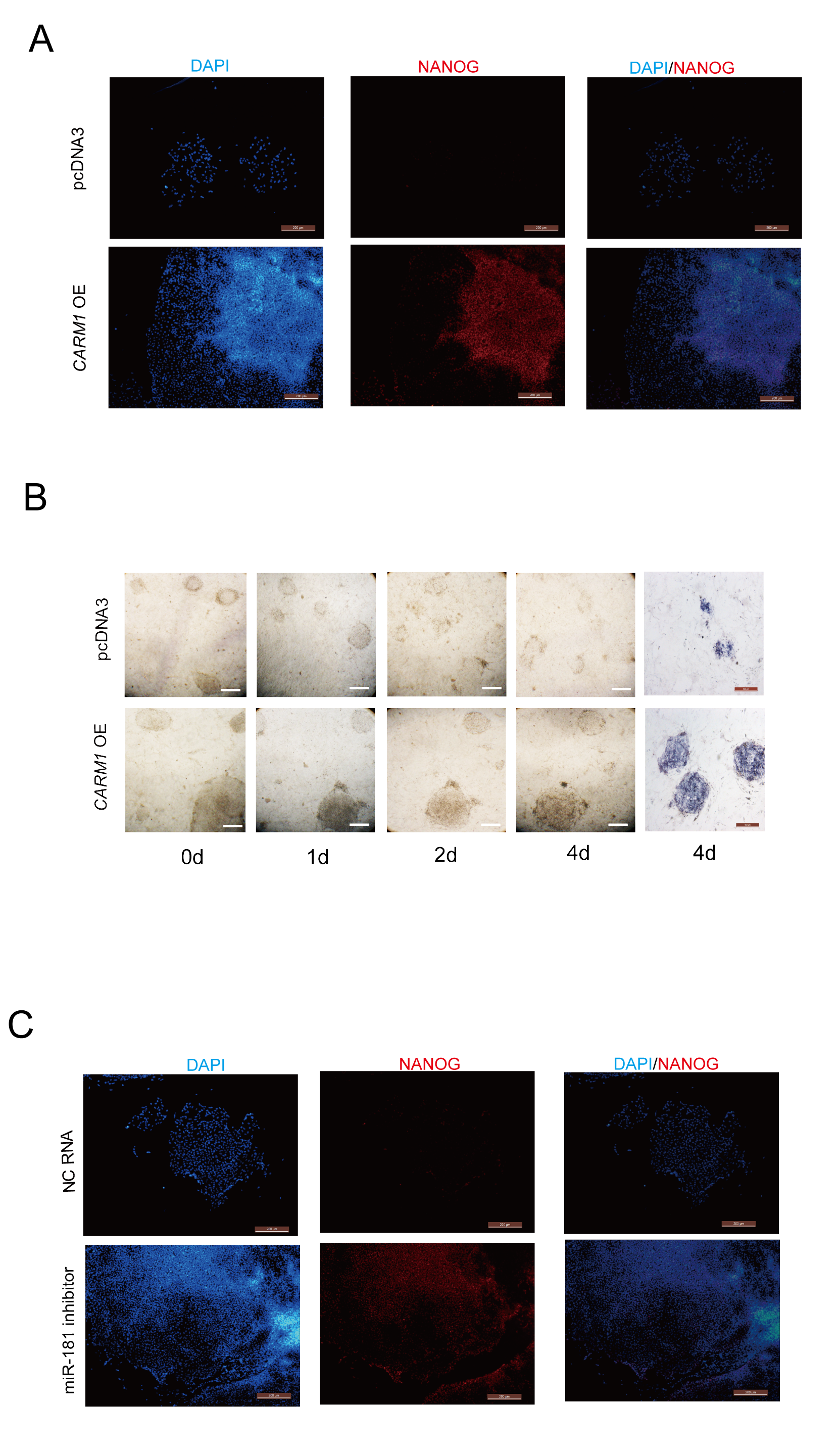

Supplement: Figure S4 — Changes of pluripotency and cell morphology in response to overexpression of CARM1 and inhibition of miR-181c upon induction of hESC differentiation. CARM1-overexpressing hESCs still expressed pluripotency markers after 8 days of BMP4-induced differentiation (A) (data for Oct4 and Sox2 not shown). CARM1-overexpressing hESCs maintained normal morphologies for as long as 4 days after the induction of differentiation (B),and their pluripotency was indicated by AP-positive colonies observed on day 4 (B). Scale bar: 200 µm in (A), 500 µm in (B). hESCs treated with miR-181c inhibitor expressed Nanog after 8 days of differentiation (C). Scale bar: 200 µm. (TIF) [file pone.0053146.s004.tif]
