# Supplementary material for: MicroRNA-181 Regulates CARM1 and Histone Aginine Methylation to Promote Differentiation of Human Embryonic Stem Cells
Source: PLoS One. 2013 Jan 3;8(1):e53146. doi: 10.1371/journal.pone.0053146 (PMC3536801; doi:10.1371/journal.pone.0053146)
Supplement: Table S1 — Oligonucleotide Sequences used in this study. (DOC) [file pone.0053146.s005.doc]

**Table S1 Oligonucleotide Sequences used in this study**

| **Gene** | **Name** | **Sequence** |
| --- | --- | --- |
| *CARM1* | Prime S | 5'-CTGGCTTTCTGGTTTGACG-3' |
|  | Prime A | 5'-CAGGAGGTTACTGGACTTGGAG-3' |
| *Nanog* | Prime S | 5'-ACCTATGCCTGTGATTTGTGG-3' |
|  | Prime A | 5'-AGTGGGTTGTTTGCCTTTGG-3' |
| *Oct4* | Prime S | 5'-GAAAGCGAACCAGTATCGAGAAC-3' |
|  | Prime A | 5'-CCCCTGAGAAAGGAGACCCA-3' |
| *Sox2* | Prime S | 5'-GGTTACCTCTTCCTCCCACTCC-3' |
|  | Prime A | 5'-CCCTCCCATTTCCCTCGTTT-3' |
| *FOXA2* | Prime S | 5'-GCCGCAGATACCTCCTACTACCA-3' |
|  | Prime A | 5'-CCCCACTTGCTCTCTCACTTGTC-3' |
| *GATA6* | Prime S | 5'-ATGACTCCAACTTCCACCTCTTCTAA-3' |
|  | Prime A | 5'-GCCCATCTTGACCCGAATACTT-3' |
| *SOX17* | Prime S | 5'-GATACGCCAGTGACGACCAGA-3' |
|  | Prime A | 5'-GCCTCGCCCTTCACCTTC-3' |
| *Brachyury* | Prime S | 5'-CTTATTTCCGTCCATTTCCCTC-3' |
|  | Prime A | 5'-GCCGCAGTAGTAGTGCTGTTCT-3' |
| *GATA2* | Prime S | 5'-GGACAGACGAAGGCAACCATT-3' |
|  | Prime A | 5'-GGGAAGCCAGAGGAGAAGAGG-3' |
| *HAND1* | Prime S | 5'-CCTATCTGGCTCTTTCTCTCTTGTC-3' |
|  | Prime A | 5'-CATCTTCCTGCGTCTGGTTCTC-3' |
| *NKX2-5* | Prime S | 5'-TCACCGTCTGTCTCCCTCACC-3' |
|  | Prime A | 5'-TGTAGAAAGTCAGGCTGGCTCAA-3' |
| *Nestin* | Prime S | 5'-CCCTTCCAGACTCCACTCCC-3' |
|  | Prime A | 5'-CCCAGCCCTCCTTTCCAG-3' |
| *PAX6* | Prime S | 5'-GCTGGCTGGCTTACTTCTTCCT-3' |
|  | Prime A | 5'-CTTTTCTCCCATTCCCACCTCT-3' |
| *SOX7* | Prime S | 5'-CACCAACGGGTCCCACAGA-3' |
|  | Prime A | 5'-GCCACTCAAGGCACAAGAAGG-3' |
| *SOX1* | Prime S | 5'-TCTCCAACTCGCAGGGCT-3' |
|  | Prime A | 5'-GGGCTCCGACTTCACCAG-3' |
| *FLK1* | Prime S | 5'-AGTGAGGAAGGAGGACGAAGG-3' |
|  | Prime A | 5'-TGACAAGAAGTAGCCAGAAGAACAT-3' |
| *PDGFB* | Prime S | 5'-CCAGAAGCCATCAGCAGCAA-3' |
|  | Prime A | 5'-CCGAGCAGGTCAGAACGAAGG-3' |
| *Zic1* | Prime S | 5'-GCCTCCCACCATCGTGTCTC-3' |
|  | Prime A | 5'-GATTCGCAGGGTTCTTTCAGTAA-3' |
| *β-actin* | Prime S | 5'- AGTTGCGTTACACCCTTTCTTG -3' |
|  | Prime A | 5'- GCTGTCACCTTCACCGTTCC -3' |
| *Pri-miR-181c/d* | Prime S | 5'-CAAGGGTTTGGGGGAACATT-3' |
|  | Prime A | 5'-GCCTCAGGGTCCACTCAACG-3' |
| *Pri-miR-181a1/b1* | Prime S | 5'-ACTGTTGTTTCTGTCTCCCATCC-3' |
|  | Prime A | 5'-GTGTCCACCTTTGGTTTCCTG-3' |
| *Pri-miR-181a2/b2* | Prime S | 5'-CAGTCTATCCCACAGTTCATTAGTTCT-3' |
|  | Prime A | 5'-CCCTTCTTCATTCTCCCATCC-3' |
| **ChIP-PCR primers** |  |  |
| *Oct4* 1stEF-1 | Prime S | 5'- GCCCCATCGGAGTTGCT-3' |
|  | Prime A | 5'- CCCGCCGTATGAGTTCTG-3' |
| *Oct4* pro-1 | Prime S | 5'- GGACTACAGGCTTGGACCACT-3' |
|  | Prime A | 5'- GTTGGGAGTTGAAAGTTGGGT-3' |
| *Oct4* pro-2 | Prime S | 5'- CACTCTTATGTTGCCTCTGTTCG-3' |
|  | Prime A | 5'- CAAAGGTGCCGTGATGGTT-3' |
| *Nanog* 1stEF-1 | Prime S | 5'-CCTCACACGGAGACTGGTAAGAAA-3' |
|  | Prime A | 5'-CTGGGACATAGTTCAAAGGGCA-3' |
| *Nanog* pro-1 | Prime S | 5'-GGACTACAGACACCCACCACC-3' |
|  | Prime A | 5'-TATGGTTCAACAGGAATGGGAT-3' |
| *Nanog* pro-2 | Prime S | 5'-ACGAGACATAGACTATCTGCCTGAA-3' |
|  | Prime A | 5'-GCACTCTGCTCCTGGGTCTG-3' |
| *Sox2* 1stEF-1 | Prime S | 5'-ATCCTGATTCCAGTTTGCCTCT-3' |
|  | Prime A | 5'-TTGTACATGCGGGCGCTG-3' |
| *Sox2* pro-1 | Prime S | 5'-CCTTTGCTACGGTTGAATGA-3' |
|  | Prime A | 5'-CCGACTAGAAGTTAGGAGACCC-3' |
| *Sox2* pro-2 | Prime S | 5'-GAGGCTTTGTTTGACTCCGTG-3' |
|  | Prime A | 5'-GGGTTAGAGGAGGATGAGATGG-3' |
| Control | Prime S | 5'- AGTTGCGTTACACCCTTTCTTG -3' |
|  | Prime A | 5'- GCTGTCACCTTCACCGTTCC -3' |
| **siRNA sequence** |  |  |
| *CARM1*-siRNA | Sense | 5'- CAGCUCUACAUGGAGCAGUdTdT -3' |
|  | Anti-sense | 5'- ACUGCUCCAUGUAGAGCUGdTdT -3' |
| Negative control | Sense | 5'-UUCUCCGAACGUGUCACGUTT-3' |
|  | Anti-sense | 5'-ACGUGACACGUUCGGAGAATT-3' |
| **Cloned primers** |  |  |
| *CARM1* ORF without 3’UTR | Prime S | 5'- AAGCTTAAGAAGGAGATATACATATGGCAGCG-3' |
|  | Prime A | 5'- ACCGGTGCGCTCCCGTAGTGCATGGTGTTG-3' |
| *CARM1* 3’UTR | Prime S | 5'-CGACGAGCTCTGGGCTTGTCATCTGCTGGAA-3' |
|  | Prime A | 5'-CCGCAAGCTTACAGGACAGCAGCAAGAGGC-3' |
