# Supplementary material for: MicroRNA-181 Regulates CARM1 and Histone Aginine Methylation to Promote Differentiation of Human Embryonic Stem Cells
Source: PLoS One. 2013 Jan 3;8(1):e53146. doi: 10.1371/journal.pone.0053146 (PMC3536801; doi:10.1371/journal.pone.0053146)
Supplement: Table S2 — Predicted miRNAs target CARM1 3′UTR. (DOC) [file pone.0053146.s006.doc]

**Table S2 Predicted miRNAs target *CARM1* 3’UTR**

| miRNA name | Conservation | Position of  *CARM1* 3' UTR | context score percentile |
| --- | --- | --- | --- |
| hsa-miR-497 | Conserved | 433-439/674-680 | 85/39 |
| hsa-miR-15a | Conserved | 433-439/674-680 | 84/39 |
| hsa-miR-15b | Conserved | 433-439/674-680 | 84/38 |
| hsa-miR-424 | Conserved | 433-439/674-680 | 84/36 |
| hsa-miR-195 | Conserved | 433-439/674-680 | 80/38 |
| [hsa-miR-16](http://www.mirbase.org/cgi-bin/mirna_entry.pl?acc=hsa-miR-16) | Conserved | 433-439/674-680 | 80/38 |
| [hsa-miR-181c](http://www.mirbase.org/cgi-bin/mirna_entry.pl?acc=hsa-miR-181c) | Conserved | 618-625 | 77 |
| [hsa-miR-103a](http://www.mirbase.org/cgi-bin/mirna_entry.pl?acc=hsa-miR-103a) | Conserved | 432-438 | 76 |
| [hsa-miR-107](http://www.mirbase.org/cgi-bin/mirna_entry.pl?acc=hsa-miR-107) | Conserved | 432-438 | 76 |
| [hsa-miR-181b](http://www.mirbase.org/cgi-bin/mirna_entry.pl?acc=hsa-miR-181b) | Conserved | 618-625 | 76 |
| [hsa-miR-181a](http://www.mirbase.org/cgi-bin/mirna_entry.pl?acc=hsa-miR-181a) | Conserved | 618-625 | 76 |
| [hsa-miR-181d](http://www.mirbase.org/cgi-bin/mirna_entry.pl?acc=hsa-miR-181d) | Conserved | 618-625 | 76 |
| [hsa-miR-4262](http://www.mirbase.org/cgi-bin/mirna_entry.pl?acc=hsa-miR-4262) | Conserved | 618-625 | 66 |
| hsa-miR-1297 | Conserved | 615-621 | 47 |
| [hsa-miR-26a](http://www.mirbase.org/cgi-bin/mirna_entry.pl?acc=hsa-miR-26a) | Conserved | 615-621 | 43 |
| [hsa-miR-26b](http://www.mirbase.org/cgi-bin/mirna_entry.pl?acc=hsa-miR-26b) | Conserved | 615-621 | 42 |
| [hsa-miR-4465](http://www.mirbase.org/cgi-bin/mirna_entry.pl?acc=hsa-miR-4465) | Conserved | 615-621 | 41 |
| hsa-miR-184 | Conserved | 652-658 | 17 |
| hsa-miR-9 | Conserved | 596-602 | 17 |
| miR-107ab | Poorly conserved |  |  |
| miR-15c | Poorly conserved |  |  |
| miR-16abc | Poorly conserved |  |  |
| miR-9ab | Poorly conserved |  |  |
| miR-322 | Poorly conserved |  |  |
| miR-1907 | Poorly conserved |  |  |
